# Stimulus-driven and behavior-driving activity along the cortical auditory hierarchy

**DOI:** 10.1016/j.neuroimage.2026.121801

**Published:** 2026-02-11

**Authors:** Kirill V. Nourski, Mitchell Steinschneider, Ariane E. Rhone, Matthew A. Howard

**Affiliations:** a Department of Neurosurgery, The University of Iowa, Iowa City, IA 52242, USA; b Iowa Neuroscience Institute, The University of Iowa, Iowa City, IA 52242, USA; c Departments of Neurology, Neuroscience, and Pediatrics, Albert Einstein College of Medicine, Bronx, NY 10461, USA; d Pappajohn Biomedical Institute, The University of Iowa, Iowa City, IA 52242, USA

**Keywords:** High gamma, iEEG, Semantic processing, Speech

## Abstract

Auditory areas on the superior temporal plane and lateral convexity are key initial stages of speech processing in the human cortex, representing acoustic and phonetic attributes in a temporally precise manner. More complex representations in auditory-related cortex along the ventral and dorsal processing streams and prefrontal cortex are associated with perception and action. In this study, we used intracranial electroencephalography (iEEG) to clarify where and how activity leading to perceptually driven behavioral events emerges. Participants were patients undergoing iEEG monitoring for medically intractable epilepsy. Stimuli were monosyllabic words, and participants pressed a button in response to a semantic target category. Significant high gamma activity after stimulus onset and immediately prior to motor response defined stimulus- and behavior-related activity patterns, respectively. The stimulus-related pattern was more common than behavior-related throughout the cortical auditory hierarchy as well as sensorimotor cortex. Behavior-related activity was sparsely represented, with the highest prevalence in the prefrontal cortex and a more limited representation in anterior temporal and parieto-occipital cortex. Hemispheric asymmetries included a higher prevalence of stimulus-related activity in the right sensorimotor cortex and a higher prevalence of the behavior-related pattern in the left prefrontal cortex. Faster behavioral responses were associated with greater stimulus-locked high gamma power in non-core auditory, prefrontal, and premotor cortex. Results reveal the cortical distribution of sensory stimulus-driven responses and activity time-locked to behavior and provide insights into neural substrates of speech perception.

## 1. Introduction

Speech communication requires that sounds be transformed into objects with semantic meaning. This process involves activation at multiple levels along the cortical auditory hierarchy, from auditory cortex, through ventral and dorsal auditory-related regions and the prefrontal cortex (Rauschecker and Scott, 2009; Chang et al., 2015). Each area along these pathways is presumed to perform distinct computations that ultimately lead to conscious perception and action (e.g., Hickok and Poeppel, 2007; Hickok, 2009).

Our previous work utilized intracranial electroencephalography (iEEG) in neurosurgical epilepsy patients to examine cortical speech processing associated with a semantic categorization task (Steinschneider et al., 2014; Nourski et al., 2017, 2021, 2022, 2024b). Stimuli were monosyllabic words associated with different semantic categories (animals/numbers/colors). High gamma activity within core auditory cortex (posteromedial portion of Heschl’s gyrus, HGPM) was primarily time-locked to word onset, robust to sound stimuli regardless of their context, and minimally modulated by task requirements (Steinschneider et al., 2014; Nourski et al., 2017). By contrast, activity within non-core auditory cortex, and in ventral and dorsal auditory-related areas could be modulated by both task and performance (Nourski et al., 2017, 2021, 2024b). These effects were especially prominent at the level of prefrontal cortex [inferior and middle frontal gyri (IFG, MFG)] in the left hemisphere (Nourski et al., 2017).

An important consideration when examining auditory processing in active tasks is that the temporal relationship between the sensory stimulus and the subsequent behavior is not rigid. Detection of a target stimulus leads to behavioral responses that are variable with respect to the timing of the stimulus. This creates a distinction between early stimulus-driven and later time-variable activity preceding behavior. It has been proposed that this later activity preceding behavior is related to conscious sensory processing (Christison-Lagay et al., 2025). Neural activity at lower levels of the cortical auditory hierarchy can be expected to follow the stimulus, while activation at higher cortical levels that supports semantic classification of the stimulus will result in temporal decoupling between the stimulus and the behavioral response (Russ et al., 2007). Studies characterizing neural correlates of auditory perception have examined responses time-locked to the task-related behavior (Sanchez et al., 2020; Sergent et al., 2021; Yu et al., 2025). Results supported the conclusion that perceptual processing manifested as an increase in neural activity immediately prior to task-related reporting.

A potential confound for examining behavior-locked activity as a measure of perceptual processing is the behavior itself (Hanks and Summerfield, 2017). Activity immediately prior to execution of a task-related behavior may, at least in part, reflect the neural commands necessary to perform the task (Cogan et al., 2014). This issue becomes especially problematic when the behavioral task requires a verbal response. The associated articulatory codes can be expected to be complex and to occur in areas such as the IFG, premotor cortex (PMC) and sensorimotor cortex that also are crucial for perceptual processing (Flinker et al., 2015; Glanz Iljina et al., 2018; Franken et al., 2022; Yu et al., 2025).

The present iEEG study sought to test the hypothesis that there is an emergence of neural activity between auditory cortex in the superior temporal gyrus (STG) and higher order areas that is time-locked not to the speech stimuli, but to subsequent behavioral responses. This behavior-locked activity is hypothesized to be more pronounced in the left hemisphere, consistent with previous work on left-lateralized dominance in the cortical processing of speech (Riès et al., 2016; Zatorre, 2022). To better dissociate perceptual processing and motor control, the behavioral output of the task was a button press rather than a verbal response. Additionally, the task required the use of the hand ipsilateral to the majority of recording sites (Nagasawa et al., 2010).

## 2. Methods

### 2.1. Participants

Participants were 49 neurosurgical patients (23 female, age 18–56 years old, median age 34 years old) diagnosed with medically refractory epilepsy who were undergoing chronic iEEG monitoring to identify potentially resectable seizure foci. Demographic, iEEG electrode coverage, and task performance data for each participant are presented in Supplementary Table 1. Forty-three participants were right-handed, four (R292, R334, R456, R717) were left-handed, and two (L416 and R764) were ambidextrous. The prefix of the participant code indicates the hemisphere with predominant electrode coverage: L for left (*N* = 26), R for right (*N* = 20), B for bilateral (*N* = 3). Forty-two participants were left hemisphere language dominant per Wada testing, two (R292, R717) were right hemisphere dominant, three (L640, L702 and L789) had bilateral language dominance. In the two remaining participants (R322, R567, both right-handed), a Wada test was not clinically warranted, and thus language dominance was not formally established.

All participants were native English speakers except for L275, a 30-year-old native Bosnian speaker who had English formal education and over 10 years of exposure. Word recognition scores, averaged between left and right ear, were 92 % in participants L275, R334, and L423, and ≥94 % in all other tested participants. Average left/right speech reception thresholds were within 20 dB HL in all tested participants. All participants underwent a preoperative neuropsychological evaluation, and no cognitive deficits that should impact the findings presented in this study were identified. Research protocols were approved by the University of Iowa Institutional Review Board and the National Institutes of Health. Written informed consent was obtained from all participants. Research participation did not interfere with acquisition of clinical data, and participants could rescind consent without interrupting their clinical evaluation.

### 2.2. Stimuli and procedure

Experimental stimuli were monosyllabic words and complex tones presented in three target detection task blocks (Steinschneider et al., 2014; Nourski et al., 2017, 2021, 2022, 2024b). The words were obtained from the TIMIT speech corpus (Garofolo et al., 1993) and LibriVox (http://librivox.org/) audiobook catalog. Words “cat”, “dog”, “five”, “ten”, “red”, and “white” were each presented 20 times per block, with each exemplar spoken by a different talker. This resulted in a total of 20 unique exemplars of each word, 14 spoken by different male and 6 by different female talkers. Additionally, blocks 2 and 3 each introduced 10 novel words each (5 target and 5 non-target, e.g. “bat”, “sit”) that were not previously presented in earlier block(s). These words, while used frequently in the English language, were unexpected in the context of the task, and thus considered novel. A detailed analysis of effects associated with this semantic novelty has been previously presented in Nourski et al. (2024b). The use of unique stimulus exemplars and additional novel stimuli were meant to promote the participants’ attention to each token and maximize their reliance on lexico-semantic cues, while minimizing reliance on phonemic cues. The complex tones included four harmonics of fundamental frequencies of 125 Hz (28 trials) and 250 Hz (12 trials), approximating fundamental frequencies of male and female talkers, respectively. Responses to the tone stimuli have been presented elsewhere (Steinschneider et al., 2014; Nourski et al., 2017, 2021, 2022). Targets were either the complex tones (block 1) or words belonging to the specific semantic categories of animals or numbers (blocks 2 and 3, respectively). At the beginning of each block, the participant was told by the experimenter which category was the target. The participant’s task was to push a button on a Microsoft SideWinder game controller (Microsoft, Redmond, WA) or a USB numeric keypad whenever they heard a target. The participants were instructed to press the button using the hand ipsilateral to the hemisphere in which the majority of electrodes were implanted. This was done to reduce contributions of preparatory, motor, and somatosensory responses associated with the button press to the recorded neural activity (c.f. Nagasawa et al., 2010).

Stimuli were presented via insert earphones (ER4B, Etymotic Research, Elk Grove Village, IL) integrated into custom-fit earmolds. The stimuli had a duration of 300 ms, were normalized to the same root-mean-square amplitude and were presented in random order with an inter-stimulus interval chosen within a Gaussian distribution [mean 2 s; standard deviation (SD) 10 ms]. Prior to each experiment, the participants were presented with a preview of a Block 1 stimulus sequence to ensure volume was at a comfortable level (typically, 55–65 dB SPL) and that the participants understood the task requirements. Experiments were carried out in a dedicated electrically shielded suite in the University of Iowa Institute for Clinical and Translational Science’s Clinical Research Unit or in the Stead Family Children’s Hospital. Experiments were carried out at least three hours after the most recent ictal event. The participants were fully awake and alert and were reclining in a hospital bed or an armchair during the experiments.

### 2.3. Recordings

Recordings were obtained using either subdural and depth electrodes, or depth electrodes alone, based on clinical requirements determined by the team of epileptologists and neurosurgeons. Details of electrode implantation, recording and iEEG data analysis have been described previously (e.g., Nourski and Howard, 2015). Electrode arrays were manufactured by Ad-Tech Medical (Racine, WI) or PMT (Chanhassen, MN). Subdural electrode arrays, implanted in 29 participants out of 49, consisted of platinum-iridium discs (2.3 mm diameter, 5–10 mm inter-electrode center-to-center distance), embedded in a silicon membrane. Stereotactically implanted depth arrays included between 4 and 14 cylindrical contacts along the electrode shaft (0.8–1.3 mm diameter), with 2.2–10 mm inter-electrode distance. A subgaleal electrode, placed over the cranial vertex near midline, was used as a reference in all participants.

Data acquisition was done by a TDT RZ2 real-time processor (Tucker-Davis Technologies, Alachua, FL) in participants L275 through L357 and by a Neuralynx Atlas System (Neuralynx, Bozeman, MT) in participants L369 through B891. Recorded data were amplified, filtered (0.7–800 Hz bandpass, 5 dB/octave rolloff for TDT-recorded data; 0.1–500 Hz bandpass, 12 dB/octave rolloff for Neuralynx-recorded data), and digitized at a sampling rate of 2034.5 Hz (TDT) or 2000 Hz (Neuralynx).

### 2.4. Analysis

Behavioral task performance was characterized in terms of hit rates (% correctly identified target words). Sensitivity was defined as $d^{\prime }=Z_{hit}-Z_{falsealarm}$, where *Z* is the inverse of the cumulative distribution function of the normal distribution. Reaction times (RT) were calculated for all hit trials relative to the onsets of the target stimuli. Hit trials with RTs below 550 ms were excluded from analysis to avoid the overlap of time windows used to estimate stimulus- and behavior-related neural activity (see below).

Anatomical localization of recording sites relied on post-implantation structural magnetic resonance imaging (MRI) and computed tomography (CT). Images were first aligned with pre-operative T1 MRI scans using linear co-registration implemented in FSL (FLIRT) (Jenkinson et al., 2002). Accuracy of electrode localization within the pre-operative MRI space was refined using three-dimensional non-linear thin-plate spline warping to correct for post-operative brain shift and distortion (Rohr et al., 2001). The warping was constrained within 50–100 control points manually selected throughout the brain, which were visually aligned to anatomical landmarks in the pre- and post-implantation scans. Locations of recording sites were transformed into a common coordinate space [ICBM152 Montreal Neurological Institute (MNI) template] in FSL and projected onto the FreeSurfer average template brain for spatial reference.

Based on results of anatomical reconstruction of electrode locations in each participant, each recording site was assigned to one of 51 regions of interest (ROIs). The ROIs were organized into groups based on an auditory-centric cortical parcellation scheme previously used to characterize cortical representation of auditory semantic novelty (Nourski et al., 2024b) and functional geometry of cortical auditory networks (Banks et al., 2023; Krause et al., 2023). The parcellation scheme used in the present study is shown in Table 1; electrode coverage in all 49 participants is summarized in Supplementary Figure 1. Assignment of sites to ROIs was based on automated parcellation of cortical gyri, implemented in the FreeSurfer software package (Destrieux et al., 2010, 2017) and confirmed by visual inspection of anatomical reconstruction data.

For recording sites in Heschl’s gyrus, delineation of the border between auditory core cortex and adjacent non-core cortex [posteromedial (HGPM) and anterolateral (HGAL) portions of Heschl’s gyrus, respectively] was based on physiological criteria (Brugge et al., 2009; Nourski et al., 2016). Recording sites were assigned to HGPM if they exhibited phase-locked responses to 100 Hz click trains characteristic of HGPM (Brugge et al., 2009). Additionally, linear correlation coefficients between averaged evoked potentials recorded from adjacent sites were examined to identify discontinuities that could reflect a boundary between HGPM and HGAL. HGPM was the sole ROI included in the auditory core ROI group. The terms “HGPM” and “auditory core”, while referring to the same recording sites, are used in this study within the context of individual ROI level and ROI group analyses, respectively. Non-core auditory cortex in the superior temporal plane (STP), including HGAL, planum temporale (PT) and planum polare (PP) were included in the STP ROI group.

The STG was subdivided into posterior and middle non-core auditory cortex ROIs (STGP and STGM), and ventral auditory-related anterior ROI (STGA) using the transverse temporal sulcus and ascending ramus of the Sylvian fissure as anatomical landmarks. The insula was subdivided into posterior (InsP) and anterior (InsA) portions (long and short insular gyri, respectively). Middle and inferior temporal gyrus were each divided into posterior, middle, and anterior ROIs (MTGP, MTGM, MTGA, ITGP, ITGM, ITGA) by dividing each gyrus into three approximately equal-length thirds. The upper and the lower bank of the superior temporal sulcus (STSU and STSL, respectively) were considered as separate ROIs based on their distinct physiological properties (Nourski et al., 2021; Murphy et al., 2022).

Anterior cingulate cortex (ACC), as identified by automatic parcellation in FreeSurfer, was considered as part of the prefrontal ROI group (i.e., separately from the rest of the cingulate gyrus). Frontal pole (FP) included fronto-marginal and transverse frontopolar gyri as defined by automatic parcellation in FreeSurfer. PMC was defined on anatomical grounds as portions of middle and superior frontal gyri immediately adjacent to the precentral sulcus. Sites localized to the subcentral gyrus by automatic parcellation were assigned to either precentral or postcentral gyrus ROI (PreCG, PostCG, respectively) based on their location with respect to the central sulcus.

Sites identified as seizure foci, characterized by excessive noise, and depth electrode contacts localized outside grey matter, were excluded from analyses. The number of recording sites used in the analyses ranged from 57 (L640) to 231 (L525) (median 136). In total, 6744 sites were studied across 49 participants.

Analysis of iEEG data was performed using custom software written in MATLAB programming environment. Recordings were downsampled to 1000 Hz and de-noised using demodulated band transform (Kovach and Gander, 2016). Analysis of iEEG data focused on high gamma event-related band power (ERBP), calculated by bandpass filtering the iEEG signal (70–150 Hz 100th order finite impulse response filter), Hilbert envelope extraction, log transform, and normalization to a prestimulus baseline (50–200 ms prior to stimulus onset). Voltage deflections *>*5 SD and high gamma band-filtered voltage deflections *>*10 SD from the within-block mean for each recording site were considered artifacts, and trials containing such deflections were excluded from further analysis.

For visualization, target hit trials were sorted by RT (motor response), followed by missed target trials. For each target hit trial with RT*>*550 ms, high gamma ERBP was averaged in two 250 ms windows: post-stimulus (50–300 ms after stimulus onset) and pre-behavior (250–0 ms before motor response). At each recording site, significance of high gamma ERBP augmentation within each of the two windows was established using one-sample one-tailed *t*-tests. Significance threshold was set at *p* = 0.05. Three patterns of activity were identified: stimulus-related (significant ERBP only in the post-stimulus window), behavior-related (significant ERBP only in the pre-behavior window), and intermediate (significant ERBP in both windows). Correction of *p*-values for multiple comparisons and adjustments of significance criteria at the single-site level were not performed to minimize false-negative results with regard to identification of responsive sites, particularly outside auditory cortex.

Prevalence of each of the three patterns was calculated separately for left and right hemisphere on the whole-brain and ROI group (auditory core, STP, STG, ventral, dorsal, limbic, prefrontal, sensorimotor) level as the percentage of sites exhibiting the pattern relative to the total number of sites. Pattern prevalence was compared between the two hemispheres using Fisher exact tests. For this analysis, corrections for multiple comparison were made using the false discovery rate approach (Benjamini and Hochberg, 1995). As sensorimotor cortex featured a significant difference in the prevalence of stimulus-related pattern between the two hemispheres (see Results), a follow-up test compared distributions of sensorimotor cortex sites along the ventral-dorsal axis between left and right hemisphere. This was done using a two-sample two-tailed *t*-test on *z*_MNI_ coordinates. Preponderance of either stimulus- or behavior-related activity – stimulus/behavior index (“SB index”) – was computed as follows:

$$SB\;index=\frac{n_{behav}-n_{stim}}{n_{behav}+n_{stim}},$$

where $n_{behav}$ and $n_{stim}$ are the number of sites within the ROI with behavior- and stimulus-related response pattern, respectively. To avoid over-interpretation of results for ROIs with extremely limited data, SB index was calculated only for ROIs that had at least five sites with either response pattern in at least two participants.

Variability of RTs across trials and participants was examined by calculating rank correlation coefficients (Spearman’s rho) between single-trial high gamma activity, calculated in the 50–300 ms post-stimulus window, and RTs. This was done for recording sites that had significant high gamma activation in the poststimulus window, for ROIs that included at least 10 such sites. At the ROI level, rho value distributions were compared to zero using one-sample two-tailed Wilcoxon signed-rank tests, with false discovery rate correction for multiple comparisons.

## 3. Results

### 3.1. Behavioral performance

The 49 participants generally exhibited good performance in the target detection task, with a median hit rate to target stimuli of 88.9 % [interquartile range (IQR) 76.4 % – 96.7 %]. This corresponds to a median of 80 hit trials (IQR 66 – 86 trials) (Supplementary Table 1). Median percentage of fast hits (RTs *<*550 ms) was 2.36 % (IQR 0.915–9.27 %); these trials were excluded from analysis of stimulus- and behavior-related high gamma activity (see below). Median sensitivity (*d*’) was 3.42 (IQR 2.70 – 4.26); grand median reaction time was 819 ms (IQR of individual participants’ median reaction times 701 – 893 ms). Two participants had over 20 false alarm (FA) responses across the two semantic categorization blocks: R416 (23 FA trials) and R376 (22 FA trials). Another six participants had between 11 and 16 FA responses, while the remaining 41 had fewer than 10. High accuracy and sensitivity exhibited by the majority of participants in this task precluded separate analyses of neural activity associated with missed or FA trials. Therefore, this study focused exclusively on neural activity associated with correct target hit trials.

### 3.2. Exemplar iEEG data

**Fig. 1.**
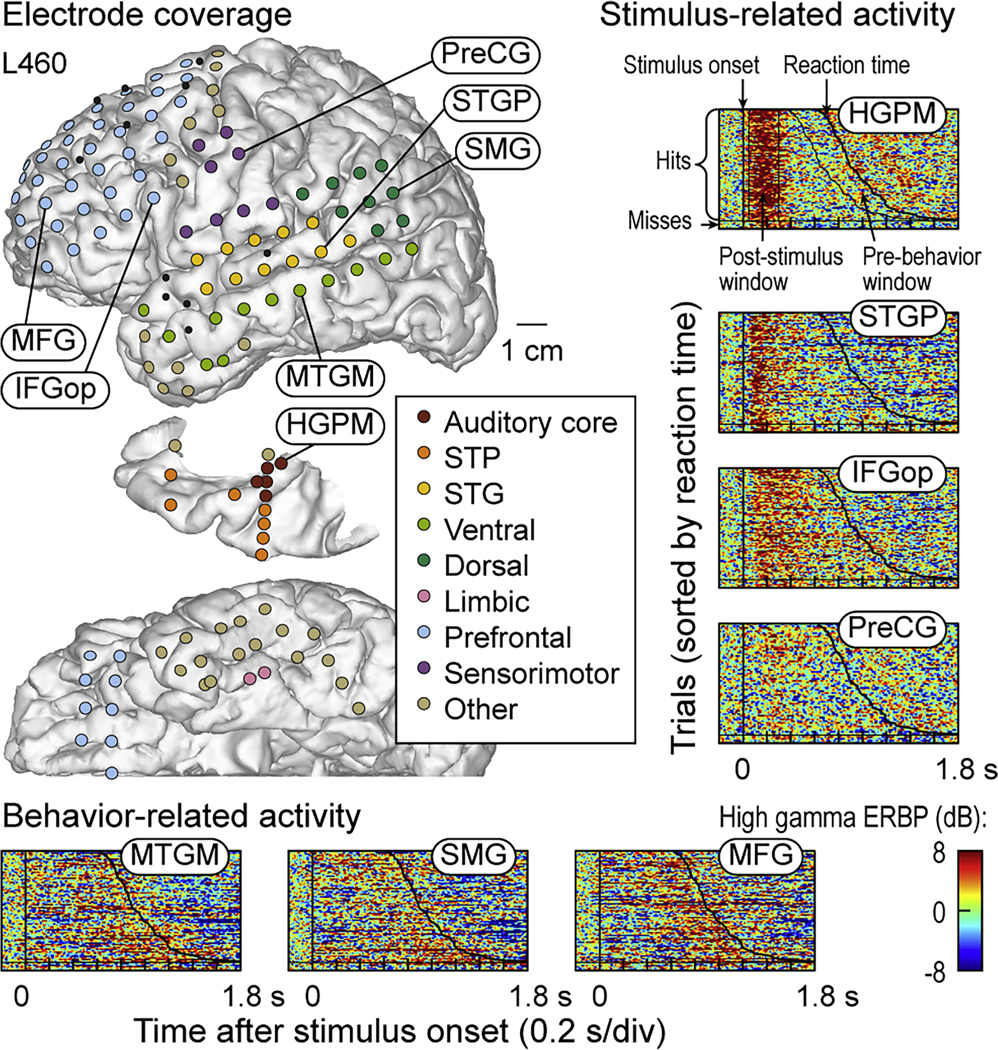
iEEG electrode coverage in an exemplar participant and waterfall plots of single-trial high gamma event-related band power (ERBP) envelopes. Electrode contact locations, color-coded by ROI group, are shown in the lateral, top-down (superior temporal plane), and ventral views. Target trials are sorted by reaction time (button press in response to the semantic target stimulus, a 300 ms monosyllabic word), followed by missed target trials (separated from hits by horizontal lines). Examples of two patterns of activity are shown. *Stimulus-related* activity is defined as significant high gamma event-related band power (ERBP) within 50–300 ms after the onset of the stimulus. *Behavior-related* activity is defined as significant high gamma ERBP within 250 ms immediately preceding the behavioral motor response (button press with left hand). HGPM: Heschl’s gyrus, posteromedial portion; IFGop: inferior frontal gyrus, pars opercularis; MFG: middle frontal gyrus; MTGM: middle temporal gyrus, middle portion; PreCG: precentral gyrus; SMG: supramarginal gyrus; STG: superior temporal gyrus; STGP: STG, posterior portion; STP: superior temporal plane.

Results are based on analysis of high gamma activity measured at 6744 recording sites across 51 ROIs in 49 participants. Neural responses to target words were characterized by stimulus-locked activity temporally aligned with the onset of the word, and behavior-locked activity preceding the motor response, temporally aligned with behavioral RTs. These response patterns are illustrated in Fig. 1. Here, electrode coverage in a representative participant is shown along with single-trial high gamma responses to target words. Subdural and depth electrode coverage included multiple cortical areas in temporal, prefrontal and sensorimotor cortex. The waterfall plots depict single-trial high gamma ERBP sorted by RT (top to bottom, shorter to longer RTs) for representative recording sites. Within auditory cortex (HGPM, STGP), high gamma activity increased following target stimulus onset and subsided prior to the button press. A similar pattern was also observed in areas outside auditory cortex (IFGop, PreCG), albeit with a longer onset latency. In this example, activity within PreCG shown was not temporally associated with the motor response. The PreCG site was located in the mid portion of the gyrus (*z*_MNI_=44.1 mm), corresponding to the representation of face within the motor homunculus (cf. Roux et al., 2020). By contrast, the exemplar sites in MTGM, SMG and MFG were characterized by high gamma activity time-locked to the subsequent behavioral response. Here, high gamma activity in trials associated with shorter RTs began earlier than activity associated with longer RTs.

To formally test for stimulus- and behavior-related activity patterns at individual recording sites, high gamma ERBP in each trial was averaged within two windows: 50–300 ms after stimulus onset (“post-stimulus window”) and 250 ms immediately preceding the button press (“pre-behavior window”). If post-stimulus, but not pre-behavior, high gamma activity was significantly greater than baseline, the pattern was defined as “stimulus-related”. By contrast, if activity within the pre-behavior, but not post-stimulus window was greater than baseline, the pattern was defined as “behavior-related”. Neural responses were only included if the two windows did not overlap in time (i.e., only hit trials with RT *>* 550 ms from the stimulus onset were included, to accommodate both analysis windows). Significant high gamma activity in both windows (an “intermediate” pattern) did not permit an unambiguous interpretation as to whether the high gamma response was primarily prolonged stimulus-related, early-onset behavior-related, or a combination of both. The intermediate pattern was not observed in the participant depicted in Fig. 1.

### 3.3. Regional and hemispheric distribution of stimulus- and behavior-related high gamma activity

**Fig. 2.**
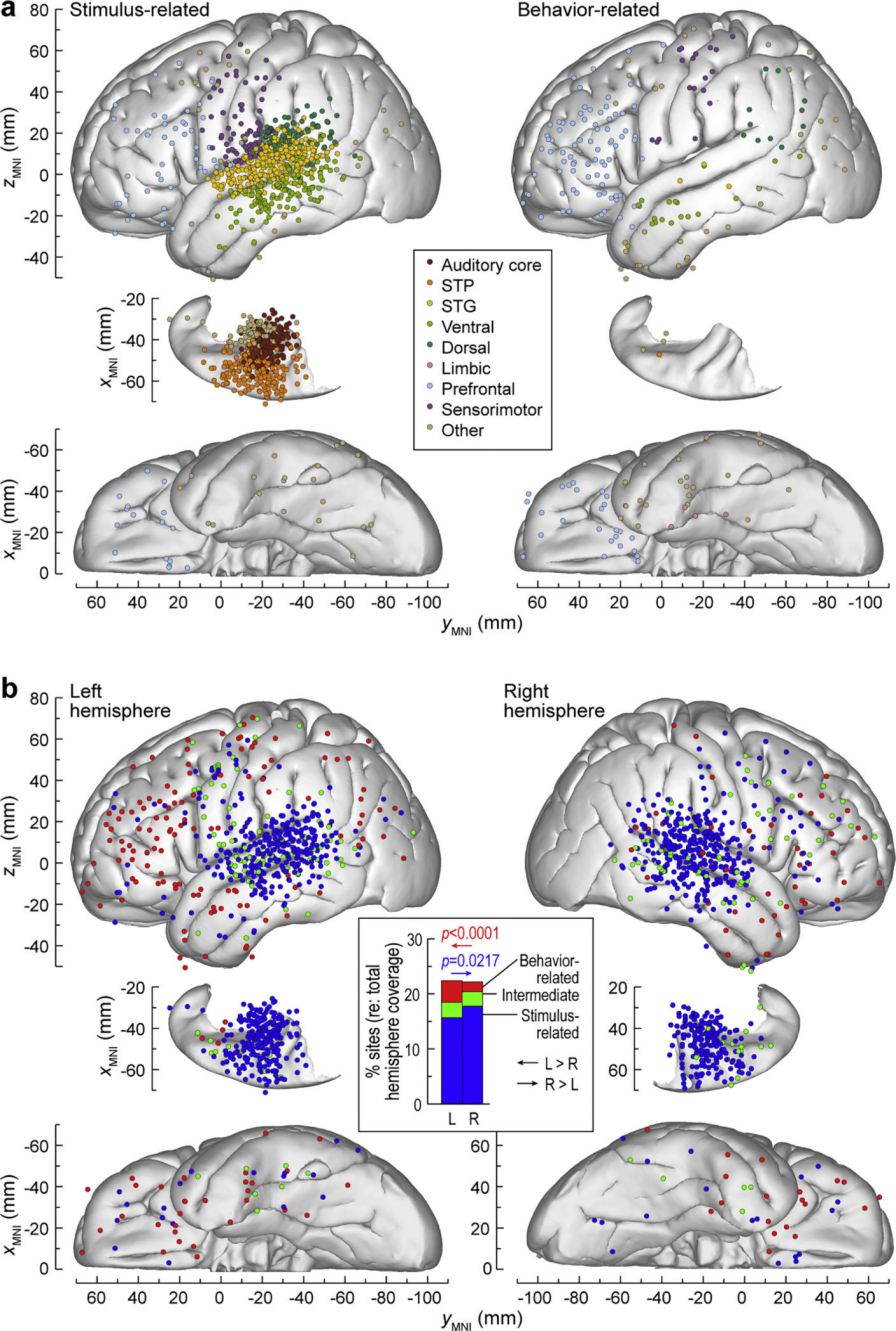
Distribution of the high gamma activity patterns across the cortex. Summary of results from 49 participants. **a:** Regional distribution of stimulus- and behavior-related activity. Sites are color-coded by ROI group and plotted in MNI coordinate space, projected onto FreeSurfer average template brain. Right hemisphere *x*_MNI_ coordinates were multiplied by (− 1) to map them onto the left-hemisphere common space. **b:** Hemispheric distribution of stimulus-related, intermediate and behavior-related pattern (blue, green and red symbols, respectively). Site locations are plotted in MNI coordinate space and projected onto FreeSurfer average template brain, separately for left and right hemisphere. **Inset:** comparison of prevalence of the three activity patterns throughout the brain between left and right hemisphere. L: left; R: right; STG: superior temporal gyrus; STP: superior temporal plane.

Topographic distribution of stimulus- and behavior-related high gamma activity is summarized in Fig. 2a (left and right panel, respectively). The stimulus-related pattern was considerably more common than behavior-related (1122 vs. 198 sites out of 6744, corresponding to an overall prevalence of 16.6 % and 2.94 %, respectively). This difference in prevalence occurred in most examined brain regions, with the exception of prefrontal and parieto-occipital cortex. Notably, the stimulus-related pattern was more common than behavior-related in sensorimotor cortex. Behavior-related activity was essentially absent in auditory cortex, including HGPM and non-core areas located on the STP and on the lateral STG.

Stimulus-related, behavior-related and intermediate pattern were also examined with respect to hemisphere (Fig. 2b). Stimulus-related pattern had an overall higher prevalence in the right hemisphere compared to the left (left: 571/3643 sites, right: 551/3101 sites, *p* = 0.0217, OR=0.860, CI 0.757 to 0.978; Fig. 2b, inset). By contrast, the behavior-related pattern was more common in the left hemisphere than in the right (left: 143/3643; right: 55/3101; *p <* 0.0001, OR=2.26, CI 1.65 to 3.10). The intermediate pattern did not exhibit an asymmetry between the two hemispheres (left: 102/3643; right: 82/3101; *p* = 0.708, OR=1.06, CI 0.790 to 1.42).

**Fig. 3.**
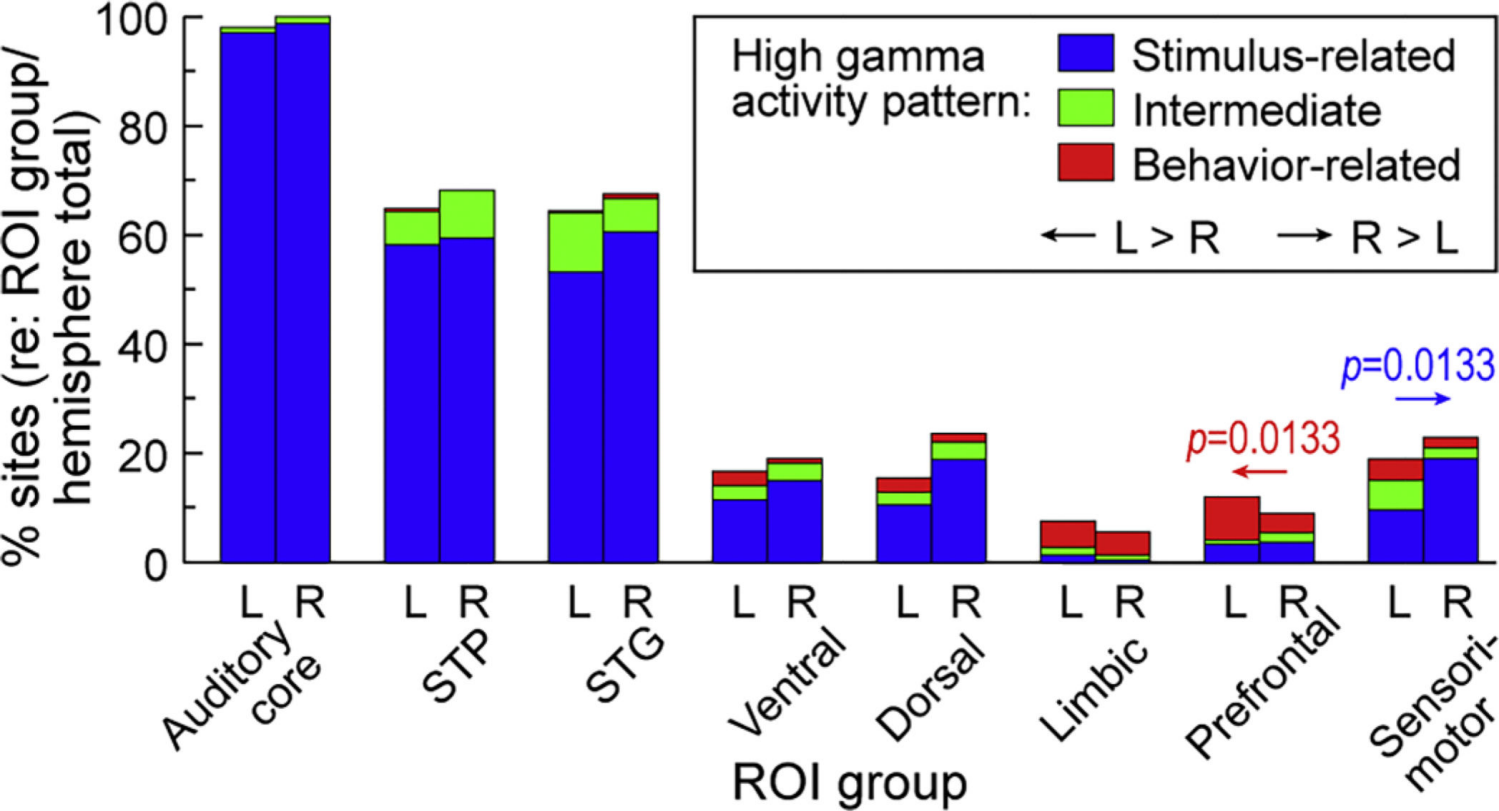
Prevalence of stimulus-related, intermediate and behavior-related pattern (blue, green and red, respectively) in the left (L) and right (R) hemisphere across region-of-interest (ROI) groups. STP: superior temporal plane; STG: superior temporal gyrus.

Hemispheric differences in the prevalence of stimulus-, behavior-related and intermediate activity patterns were further examined at the level of the eight ROI groups, from auditory core to sensorimotor cortex (Fig. 3, Table 2). A significantly higher prevalence of stimulus-related activity in the right hemisphere was noted in the sensorimotor ROI group (*p* = 0.0133, OR=0.4528, CI 0.280 to 0.73). Of note, there was a small but significant difference in the distribution of recording sites localized to sensorimotor cortex along the *z*_MNI_ (i.e. ventral-dorsal) axis. That is, the coverage in the right hemisphere had a more ventral distribution than on the left (mean *z*_MNI_ 36.4 mm and 32.6 mm in the left and right hemisphere, respectively; *p* = 0.0157, *t* = 2.42). The higher prevalence of the stimulus-related pattern in the right hemisphere could in part reflect this coverage bias. The behavior-related pattern, on the other hand, was more common in the left prefrontal cortex than in the right (*p* = 0.0133, OR=2.35, CI 1.45 to 3.81). Overall, there was a progressive reduction in the prevalence of stimulus-related activity and an increase in the prevalence of behavior-related activity along the auditory processing hierarchy.

### 3.4. Patterns of neural activity across the cortical auditory hierarchy

The previous section described hemispheric asymmetries for prevalence of stimulus- and behavior-related activity. To examine these asymmetries at the ROI level, high gamma stimulus/behavior (SB) index was calculated for individual ROIs as the difference between the numbers of sites with behavior-related and stimulus-related activity divided by their sum. An SB index of − 1 indicates an ROI where stimulus-related, but not behavior-related activity, was present. SB index = 1 indicates behavior-related activity in the absence of stimulus-related activity. SB index = 0 denotes an equal distribution of the two activity patterns. For each hemisphere, only ROIs that had at least five sites exhibiting either pattern in at least two participants were included to avoid over-interpretation of results.

**Fig. 4.**
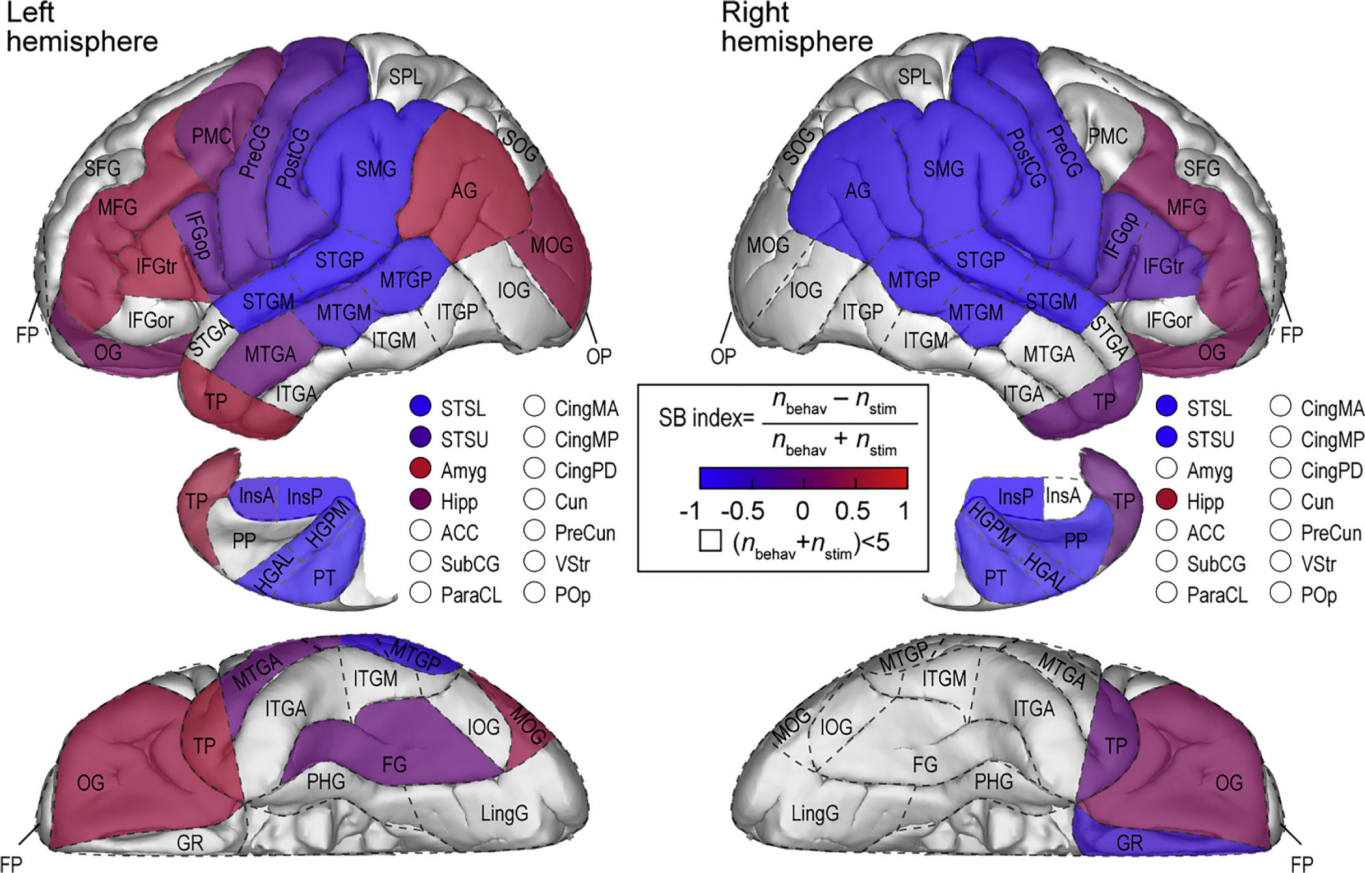
High gamma stimulus/behavior (SB) index. The SB index was calculated for ROIs with at least five sites exhibiting either pattern in at least two participants, separately for left and right hemisphere (see Table 3). Blue indicates an ROI where stimulus-related activity was more common than the behavior-related pattern, red indicates the opposite relationship. ROIs that did not meet the inclusion criteria are not colored. See Table 1 for ROI abbreviations.

High gamma activity in the auditory cortex (HGPM, STP, STG) in both hemispheres was almost exclusively stimulus-related (SB index *<* − 0.95) (Table 3 and Fig. 4). Similar values were observed in InsP (left: − 0.895, right: − 1). Several ROIs along ventral (MTGM, MTGP, STSU, STSL) and dorsal (SMG) auditory cortical pathways also exhibited activity predominantly time-locked to the stimulus. The behavior-related pattern, present at a minority of sites in these ROIs, was more common in the left hemisphere. There was a further shift in SB index to less negative values in the IFGop and sensorimotor cortex (PreCG, PostCG) – again, more prominent in the left hemisphere.

In the left hemisphere, several other ROIs had the SB index weighted towards behavior-related activity: AG, amygdala, hippocampus, IFGtr, MFG, OG, MOG, PMC, TP. In the right hemisphere, this bias for behavior-related activity was observed in the hippocampus, MFG, and OG. By contrast, right AG and right IFGtr had a stronger bias towards stimulus-related activity, while the right TP had a SB index = 0. IFGor in both hemispheres and amygdala, MOG, and PMC in the right hemisphere all had insufficient number of responsive sites to establish the SB index. Likewise, the number of sites in the subregions of the right anterior temporal lobe (STGA, MTGA, ITGA) was insufficient to characterize each ROI in terms of asymmetries of the stimulus-and behavior-related activity. The SB index values calculated for the TP (left: 0.667, right: 0) were representative of the anterior temporal lobe as a whole (i. e., including TP, STGA, MTGA and ITGA) (left: 0.400, right: 0.0769). In summary, there was an emerging behavior-related activity in auditory-related regions, with a greater representation of this pattern in anterior temporal, parieto-occipital and prefrontal regions, particularly in the left hemisphere.

### 3.5. Timing of stimulus-locked activity across ROIs

**Fig. 5.**
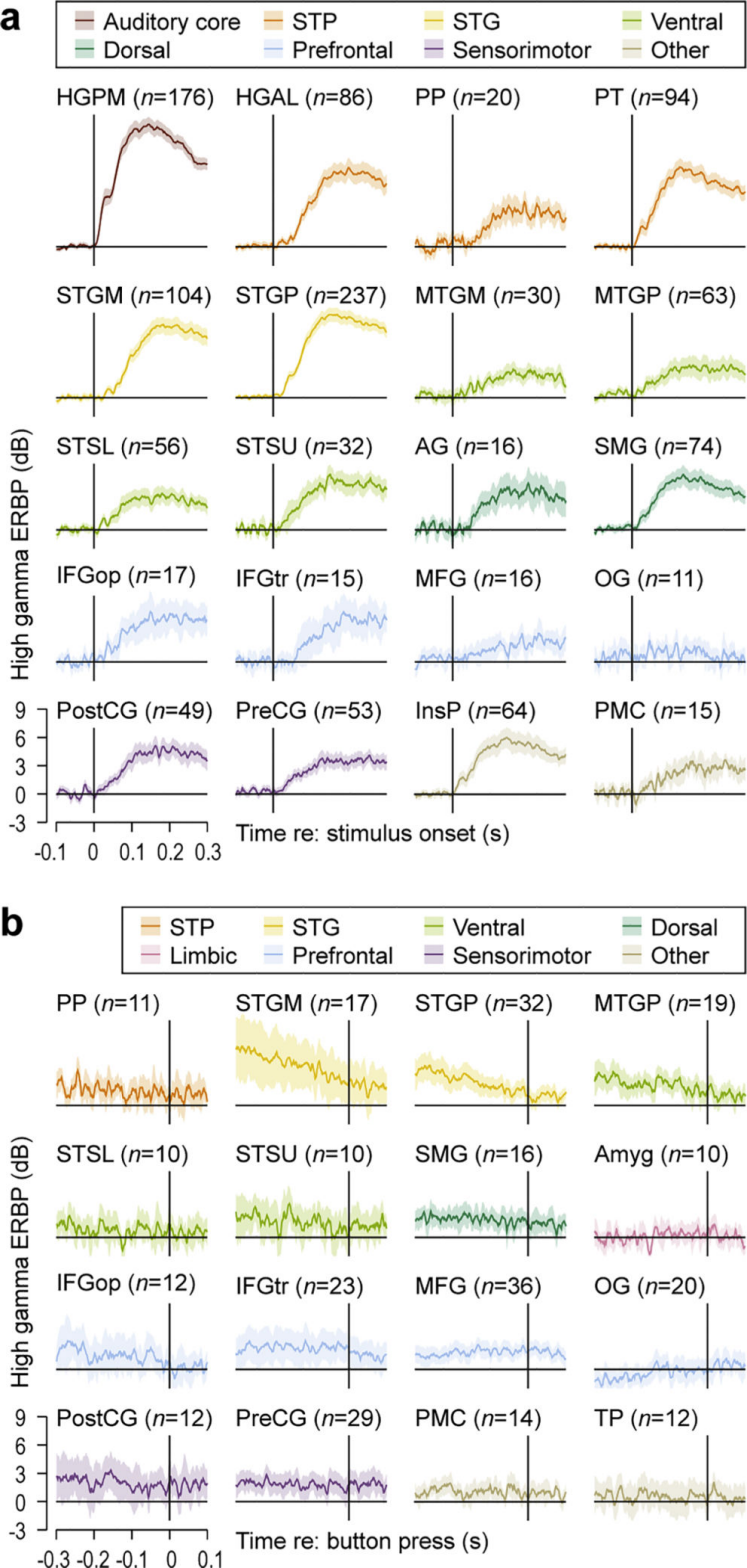
Timing of stimulus- and behavior-locked activity across regions of interest (ROIs). **a:** High gamma event-related band power (ERBP) envelopes, averaged across recording sites with stimulus-related or intermediate activity pattern, for each ROI that included at least 10 such sites. ERBP is plotted as a function of time relative to stimulus onset. **b:** High gamma ERBP envelopes, averaged across recording sites with behavior-related or intermediate activity pattern, for each ROI that included at least 10 such sites. ERBP is plotted as a function of time relative to button press. Plots are color-coded by ROI group. Lines and shading represent across-site means and 95 % confidence intervals, respectively. Sites characterized by intermediate pattern (significant high gamma ERBP in both post-stimulus and pre-behavior window) are included in both panels. See Table 1 for ROI abbreviations.

As previously discussed, stimulus-related activity was prominent throughout the cortical auditory hierarchy, beginning in HGPM and extending into prefrontal and sensorimotor areas. To examine the time course of this activity, high gamma ERBP envelopes, measured between − 100 and 300 ms relative to stimulus onset, were averaged across trials for each recording site with significant activity in the post-stimulus window (50–300 ms) (i.e., sites described as “stimulus-related” and “intermediate” in Fig. 2b). These ERBP envelopes were then averaged across sites for each ROI that included at least 10 sites characterized by stimulus-related or intermediate activity pattern. The resultant high gamma envelopes represent the overall time course of stimulus-locked activity within an ROI (Fig. 5a). There was considerable overlap in the time course of this activity, with multiple ROIs exhibiting concomitant activation that extended from auditory cortex along ventral and dorsal pathways into prefrontal and sensorimotor areas. This similarity of temporal activation profiles indicates extensive parallel processing distributed across the cortical auditory hierarchy.

A complementary analysis focusing on behavior-locked activity is presented in Fig. 5b Several key findings are evident. Average responses in the pre-behavior time window were generally weaker compared to those in the post-stimulus window, and their time course was more variable across ROIs. Lower stages of the cortical auditory hierarchy (STGM, STGP, MTGP) featured a steady decline in ERBP over the course of the pre-behavior window. IFGop and IFGtr featured elevated ERBP that declined to zero following the button press. By contrast, elevated ERBP within MFG, PostCG and PreCG had a relatively flat profile and persisted beyond the button press. Some ROIs (Amyg, OG, TP) had near-zero high gamma envelopes, a result of averaging ERBP with variable timing across individual sites. Of note, there was no elevation in ERBP following the button press in PostCG. Overall, this analysis did not reveal a behavior-locked neural response signature common to all sites with significant high gamma responses in the pre-behavior window.

### 3.6. Relationship between stimulus-related high gamma activity and RTs

**Fig. 6.**
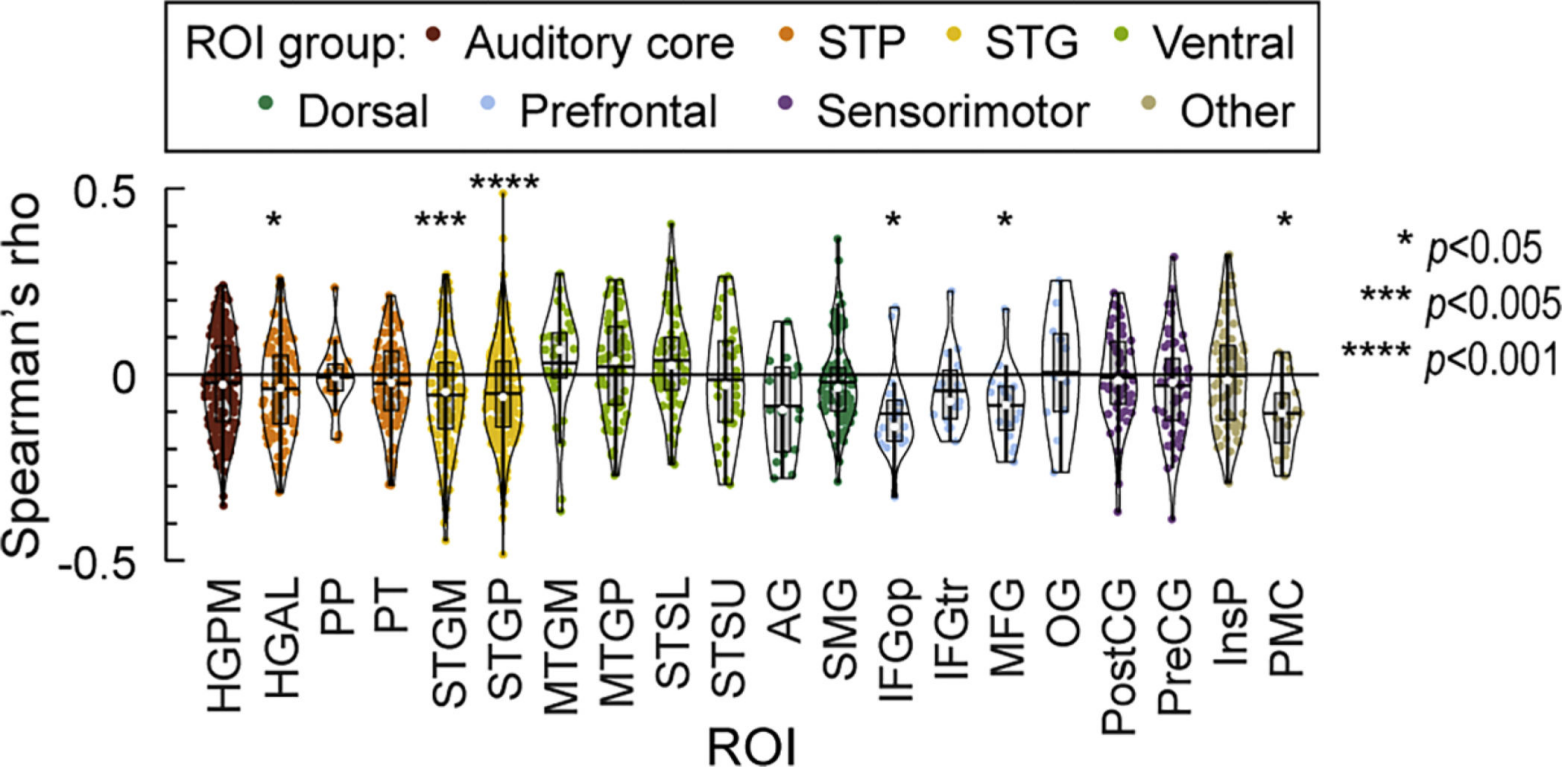
Relationship between stimulus-locked high gamma activity and RTs. Rank correlation coefficients (Spearman’s rho) between post-stimulus high gamma ERBP and RTs are plotted for recording sites with stimulus-related and intermediate activity pattern, for each ROI that included at least 10 such sites. Violin plots are color-coded by ROI group. Negative values represent an association wherein greater high gamma activation is followed by faster behavioral responses. In violin plots, white circles denote medians, horizontal lines denote means, grey boxes denote *Q*_1_ and *Q*_3_, and whiskers show the range of lower and higher adjacent values (i.e., values within 1.5 interquartile ranges below *Q*_1_ or above *Q*_3_, respectively). See Table 1 for ROI abbreviations.

The paradigm used in the present study did not impose a delay between presentation of a stimulus and the resultant behavioral response. As a result of this design, RTs varied considerably both within and across participants (see Fig. 1 and Supplementary Table 1, respectively). To examine possible sources of this variability, rank correlation coefficients (Spearman’s rho) were calculated between single-trial high gamma activity in the 50–300 ms poststimulus time window and RTs. This analysis was performed for recording sites that had significant high gamma activation in the post-stimulus window (blue and green symbols in Fig. 2b). Fig. 6 presents distributions of rho values for ROIs that included at least 10 recording sites with significant high gamma activity in the post-stimulus window. Here, negative rho values correspond to a relationship wherein greater post-stimulus high gamma activation is associated with shorter RTs. At the ROI level, the center of the distribution of rho values was significantly below zero in HGAL, STGM, STGP, IFGop, MFG and PMC (Wilcoxon signed-rank tests, false discovery rate-corrected for multiple comparisons) (Table 4). By contrast, no ROIs exhibited the opposite relationship between high gamma activity and RTs (i.e. larger high gamma activity associated with longer RTs). Thus, the magnitude of stimulus-locked activity within non-core auditory cortex, prefrontal cortex and PMC, but not in auditory core, auditory-related, and sensorimotor cortex, may have contributed to speed of behavioral responses in this task.

## 4. Discussion

### 4.1. Summary of findings

Results reveal progressive stages of cortical auditory processing that include target word-driven responses and activity that is time-locked to subsequent behavior. High gamma activity within auditory cortex is almost exclusively time-locked to the words. Higher-order areas are characterized by the emergence of activity time-locked to the button-press behavior. This emergence is more prominent in the left hemisphere, particularly in the prefrontal cortex. Surprisingly, the stimulus-related pattern is more common than that related to motor behavior in sensorimotor cortex.

### 4.2. Regional distribution of stimulus- and behavior-related high gamma activity

The preponderance of stimulus-related activity within canonical auditory cortex is consistent with its known physiological properties that include the decoding of acoustical and phonological attributes of speech (Oganian and Chang, 2019; Yi et al., 2019; Hamilton et al., 2021). A minority of auditory cortical sites displayed an intermediate response pattern, with significant increases in high gamma activity in both post-stimulus and pre-behavior time window. At this level in the hierarchy, the intermediate pattern likely reflects prolonged activation triggered by the stimulus that persists into the time period used to assess activity related to the behavioral response. This interpretation is consistent with previous studies demonstrating auditory cortical responses extending beyond several hundreds of milliseconds during passive listening when no overt behavioral response is linked to the stimulus (Steinschneider et al., 2011; Khalighinejad et al., 2017). More recent iEEG work demonstrates variable windows of integration across the auditory cortex that may be associated with prolonged stimulus-driven neural responses (Norman-Haignere et al., 2025). Pre-perceptual processing at the level of the auditory cortex is further supported by preserved local deviance detection in sedated and anesthetized states (Nourski et al., 2018, 2025).

The emergence of the later behavior-related pattern outside canonical auditory cortex likely reflects to varying degrees the lexical and semantic processing required for target detection, decision making, motor planning and performance of the subsequent behavior signaling that detection. That is, auditory-related areas along the ventral and the dorsal processing streams become not simply responsive to acoustical and phonological attributes of speech, but to task parameters and higher-order linguistic features, such as encoding phrasal meaning (e.g., Murphy et al., 2022) and semantic encoding at the sentence level (Friederici et al., 2000; 2003; Humphries et al., 2005).

MTGP and MTGM are early stages of the ventral auditory processing pathway that can feature activity time-locked to the stimulus and, to a lesser degree, to behavior. These regions are important for mapping sound onto meaning (Hickok and Poeppel, 2007; Binder et al., 2009; Kocagoncu et al., 2017). Supporting evidence includes auditory comprehension deficits following lesions affecting MTGP/MTGM (Bates et al., 2003 Turken and Dronkers, 2011). These deficits have been interpreted to reflect impairments in lexico-semantic rather than phonemic-level processing (Chertkow et al., 1997; Hart and Gordon, 1990; Dronkers et al., 2004). Cortical stimulation and functional neuroimaging results corroborate the involvement of the MTG in lexical-semantic processing (Miglioretti and Boatman, 2003; Rissman et al., 2003; Rodd et al., 2005). Finally, MTGP has been posited to be particularly important for word-level comprehension (Dronkers et al., 2004), in keeping with the task requirements of this study. Given these considerations, it is reasonable to interpret stimulus-locked activity within MTGP and MTGM as related to lexical access, and the later behavior-locked activity as related to subsequent semantic analysis. Similar arguments can be made for processing within the anterior temporal lobe (TP+STGA+MTGA+ITGA), where bilateral atrophy as seen in fronto-temporal dementia of the semantic type is associated with severe deficits in naming and single-word comprehension tasks (Amici et al., 2006; Spitsyna et al., 2006; Gorno-Tempini et al., 2004, 2011; Hodges and Patterson, 2007).

A small minority of sites within the SMG exhibited responses time-locked to the button press. The behavior-related pattern was observed in only two out of 169 sites in the left SMG and three out of 148 sites in the right SMG. This contrasts with stimulus-related activity found in 23.1 % (39/169) sites in the left SMG and 24.3 % (36/148) in the right SMG. Several explanations are possible for this difference. The SMG and the adjacent temporo-parietal junction are proximal regions of the dorsal auditory cortical pathway that are important for speech production, audiomotor control and phonological processing (Hickok, 2009; Steinschneider, 2013; Kocagoncu et al., 2017; Wandelt et al., 2024). Strokes in this region are associated with conduction aphasia and deficits in phonological processing (Damasio and Damasio, 1980; Baldo et al., 2008; Perron et al., 2024). The behavioral responses in the current study did not include generating vocalizations. Thus, it is likely that more sites would have exhibited behavior-locked activity had the task involved speaking. The sizable number of sites in the SMG active during the post-stimulus time window is consistent with its role in phonological processing. In contrast to SMG, adjacent AG exhibited a pronounced hemispheric asymmetry, with predominant behavior-related pattern in the left hemisphere and stimulus-related activity in the right (SB index 0.667 and − 0.846, respectively). Thus, high gamma activity as reported here augments the conclusion obtained from meta-analyses of functional neuroimaging studies that the left AG contributes to semantic processing (Binder et al., 2009; Price, 2012; Bemis and Pylkkänen, 2013).

The left IFG is a key component in the circuits responsible for both speech perception and production (Hullett et al., 2025; Yu et al., 2025). In addition to its major role in articulatory planning (Flinker et al., 2015), it is considered crucial for sentence-level processing including perception of syntax (Dronkers et al., 2004; Hickok, 2009; Friederici, 2011; Woolnough et al., 2023). In the current study, the IFG was also activated by single words, and both IFGop and IFGtr were engaged in the task. Behavior-related activity preceding the button press occurred at 8.45 % (6/71) and 14.3 % (14/98) sites in the left IFGop and IFGtr, respectively, whereas this response pattern was observed in only 4.88 % (2/41) 3.37 % (3/89) of sites in the right IFGop and IFGtr, respectively. Sampling was limited in IFGor, precluding a meaningful comparison with the other two divisions of the IFG. The difference in the roles of IFGop and IFGtr is supported by the observation that stimulus-related activity occurred at 11.3 % (8/71) and 12.2 % (5/41) of sites in the left and right IFGop, respectively, compared to 3.06 % (3/98) and 6.74 % (6/89) of sites in the left and right IFGtr, respectively. Multiple pathways contribute to IFG inputs from auditory cortex (reviewed in Chang et al., 2015). Given the relatively early (50–300 ms) time window by which we defined stimulus-locked activity, it is noteworthy that recent data demonstrate short latency high gamma responses in IFG to speech emanating from core auditory cortex and the medial geniculate nucleus (Hullett et al., 2025). These responses could be elicited by low-level spectrotemporal sound attributes typically associated with auditory cortical activity on the STP. Thus, stimulus-related responses we observed in the IFG may in part be driven by inputs from low level regions in the auditory cortical hierarchy and thalamus.

The MFG stands out as the region with the most prominent behavior-related responses when compared to the stimulus-related pattern predominant elsewhere. In the left MFG, behavior-related activity was found at 10.1 % (22/318) of sites whereas stimulus-related responses occurred only at 2.75 % (6/318) of sites. In the right MFG, behavior-related activity likewise was twice as common than stimulus-related (8 vs 4 sites out of 194). Current findings are consistent with a study that examined iEEG activity during an auditory naming task, which identified portions of the MFG and IFG as participating in semantic processing with a left hemisphere bias (Yu et al., 2025). A complementary iEEG study examined perception of environmental sounds presented at threshold intensity (Christison-Lagay et al., 2025). Stimuli that were perceived by the listener elicited early activation within right caudal MFG and triggered a sequence of activation spreading from auditory cortex to IFG, MFG and SMG bilaterally. By contrast, sounds that were not perceived only activated the auditory cortex. In our study, the early activity in the MFG would have been included in the post-stimulus time window, thus contributing to the stimulus-related pattern. The right hemisphere bias for the early activation within the MFG reported by Christison-Lagay et al. (2025) is opposite to that seen in the Yu et al. (2025) study; this difference may in part based on differences in the type of stimuli used (environmental sounds vs. words).

While speech-related activation of sensorimotor cortex is well established, the underlying tenets remain a contentious issue (e.g., Scott et al., 2009; Shtyrov et al., 2014; Cheung et al., 2016). One viewpoint is that speech perception requires transformation of acoustic attributes of speech into their intended phonetic gestures by the motor system (Liberman and Mattingly, 1985; Pulvermüller et al., 2006; D’Ausilio et al., 2009; Schomers et al., 2015). The other major viewpoint emphasizes that activation of sensorimotor cortex is based on processing acoustic speech attributes irrespective of their articulatory signatures (Cheung et al., 2016). In either case, strokes or other lesions of the sensorimotor cortex do not significantly impair speech perception, supporting the interpretation that activation of sensorimotor cortex during listening to speech likely plays an auxiliary role in phonetic perception (Hickok, 2009). Activity preceding articulation has typically been related to motor planning or the actual production of speech (Cogan et al., 2014; Yu et al., 2025). In the present study, activation of PreCG and PostCG occurred in both the stimulus-related and behavior-related time windows. Of note, the highest prevalence of stimulus-related activity − 27.2 % (28/103) - was in the right PostCG. Short onset latency activity measured within the stimulus-related window is in keeping with previous studies (e.g., Cogan et al., 2014; Shtyrov et al., 2014).

It may appear unusual for PostCG to be strongly activated when listening as opposed to the expected activation based on somatosensory feedback associated with speaking (Franken et al., 2022). However, it has been proposed that this activation when listening reflects attempts to match acoustic inputs with learned audio-motor or audio-somatosensory association representations (Brefczynski-Lewis and Lewis, 2017; Franken et al., 2022). Our findings cannot unequivocally determine if the stimulus-related activity was engaged in either acoustic or audiomotor processing. On the other hand, the use of a button press rather than a verbal response to report a target stimulus mitigates against contribution of articulatory planning to behavior-related activity and instead supports contribution of sensorimotor cortex to auditory processing.

### 4.3. Progressive nature of behavior-locked activity

Current findings demonstrate that there is a progressive increase in the prevalence of behavior-related neural activity from the MTG, through the anterior temporal lobe and into prefrontal cortex. Several interpretations of the behavior-related pattern can be proposed. First, this activity could be directly engaged in planning and execution of the button press. This explanation, while feasible, appears unlikely to be the main contributor to the behavior-locked activity. It would imply that activity in the temporal lobe outside of canonical auditory cortex is responsible for generating the commands necessary for making the button press. While cortical activity related to motor planning and execution is not limited to the contralateral hemisphere (Bundy and Leuthardt, 2019), motor effects are reduced when subjects use the hand ipsilateral to the recording sites, mitigating a purely motor-related interpretation (Nagasawa et al., 2010). On the other hand, if verbal responses were required to perform the task, then the behavior-locked neural activity could in part represent the commands needed for guiding the sequence of articulations associated with speech output (Yu et al., 2025).

A more likely explanation for the behavior-related activity pattern in the current study is that it reflects a progression in levels of analysis necessary for task-related identification of the target words. This interpretation is based on the hypothesis that the canonical auditory cortex and adjacent auditory-related areas maintain a greater emphasis on stimulus attributes of the words at acoustic, phonetic and lexical levels, whereas higher-order regions such as the anterior temporal lobe and prefrontal cortex are engaged to a greater extent in the identification and categorical representation of the task-relevant stimuli (Russ et al., 2007; Hickok, 2009). A corollary of this interpretation is that representation of the target words is not stored within a particular brain region, but instead is widely distributed across temporal, sensorimotor and prefrontal areas. The progressive increase in the behavior-related pattern suggests that there is a build-up of activity along the auditory hierarchy related to the task. This interpretation is in keeping with non-human primate evidence for complementary (synergistic) encoding of prediction error associated with sounds presented in the setting of oddball paradigms (Gelens et al., 2024). Synergistic activation was identified between auditory and frontal cortices utilizing strong, long-distance feedforward and feedback connections. In the current study, it is reasonable to hypothesize that the temporal lobe provides feedforward information regarding word attributes while prefrontal areas provide feedback information pertaining to target-related categorization (Russ et al., 2007).

### 4.4. Hemispheric asymmetries

Opposite hemispheric asymmetries were observed for stimulus- and behavior-related activity. At the whole cortical level, stimulus-related activity was more prevalent in the right hemisphere, whereas behavior-related activity was more prevalent in the left hemisphere. At a finer scale, stimulus-related activity was more prominent in the right sensorimotor cortex compared to the left. This interesting observation is contrary to classic descriptions of the dorsal audiomotor pathway, which typically is envisioned to be strongly left-lateralized (Hickok and Poeppel, 2007, but see Cogan et al., 2014). However, evidence including our own has accumulated to support a role of the right sensorimotor cortex in speech perception (Hickok and Poeppel, 2007; Nourski et al., 2024a, 2024b). The present finding that behavior-related activity was more prevalent in the left prefrontal cortex is consistent with other work demonstrating lateralization of semantic processing to the left IFG and MFG (Yu et al., 2025). It is of note that in the latter study, responses reflecting semantic processing were measured in the pre-articulatory window of − 500 – 0 ms, similar to the manner in which we assessed behavior-related activity in the present study.

### 4.5. Timing of neural activity: from sensory encoding to conscious perception and behavior

High gamma activity time-locked to the stimulus is interpreted to primarily reflect neural events related to sensory encoding. By contrast, it is more difficult to assign a sole interpretation to behavior-locked activity, as it can reflect multiple different processes, including conscious perception, task-related decision making and motor preparation. All these processes are components of behavior in this task and may be expected to co-occur in the pre-behavior window. In one example of the differences between stimulus- and behavior-related activity, participants were asked to report when they heard a sound presented at a near-threshold intensity by either performing or withholding a button press (Sanchez et al., 2020). When the sounds were perceived, they elicited both early magneto- and electroencephalographic responses and later activity that preceded the behavioral report. By contrast, when the sounds were not perceived, only early activity was present. The authors concluded that the early activity was associated with sensory processing, while late activity was interpreted as a manifestation of conscious perception. A complementary electroencephalography study demonstrated that beginning at about 250–300 ms after stimulus onset there is a bifurcation in neural activity (Sergent et al., 2021; see also Noel et al., 2018), wherein late sustained activity is associated with task-related reports and conscious perception, a finding parsimonious with the current data. Absence of this late activity was associated with sensory processing that did not reach a conscious level.

Results of iEEG studies provide additional support for the sequence of events posited to occur during the current semantic classification task. Perception of environmental sounds presented at near-threshold intensity was associated with widespread high gamma activity (Christison-Lagay et al., 2025). Neural responses associated with sounds that were not perceived were limited to auditory cortex and immediately adjacent areas (cf. Nourski et al., 2018, 2025). Cogan et al. (2014) reported a highly synchronized neural response spread out across multiple ROIs from the auditory cortex, and through temporal, prefrontal and sensorimotor regions. Extensive temporal overlap of responses across multiple ROIs reported by Cogan et al. (2014) is similar to that identified in the current study (see Fig. 5a) and the iEEG study by Christison-Lagay and colleagues (2025). One consequence of widespread synchrony is the opportunity for binding of the various attributes that each ROI provides computations for, from acoustic to lexical, semantic, and audiomotor.

Average responses in the pre-behavior time window were generally weaker compared to those in the post-stimulus window (see Fig. 5b) and featured a more variable time course across ROIs. Lower stages of cortical auditory hierarchy (STGM, STGP, MTGP) that featured a steady decline in ERBP had more sites with intermediate than behavior-related pattern (cf. Fig. 3). This time course likely reflects prolonged activation triggered by the stimulus that persists into the pre-behavior time period. The lack of further increase in ERBP following the button press in PostCG suggests that sensory feedback from the motor response was not a major contributor to the measured activity.

In a study by Yu et al. (2025), high gamma activity was recorded during an auditory naming task (e.g., “name an animal that says meow”) and contrasted with that associated with a repetition task (e.g., hear ‘cat’, speak ‘cat’). Relevant results include the demonstration of semantic processing in IFG and MFG that was strongly left hemisphere-dominant and increased activity prior to articulation when the task was naming compared to repetition. The latter finding supports our contention that behavior-related activity is associated with semantic processing as a prerequisite for behavior as opposed to being strictly related to motor planning and execution. In summary, the key findings of the current study align with previous iEEG and non-invasive work reporting synchronous widely distributed stimulus-locked activation across multiple tasks.

While a detailed discussion of models of conscious auditory perception is beyond the scope of this paper, several comments regarding how current data fit within some of their theoretical constructs can be made (Mashour et al., 2020; Seth and Bayne, 2022). The key starting point is the finding that multiple ROIs are active in parallel (see Fig. 5a) when a target word is identified. The global neuronal workspace theory of consciousness posits that sensory inputs reach consciousness when information emanating from sensory cortex is disseminated to multiple higher-order areas (Dehaene et al., 2014; Brodbeck et al., 2022). This dissemination, termed “ignition”, leads to the coherent activation of a subset of neurons in the global workspace (Mashour et al., 2020). Parallel activation of multiple nodes in the auditory hierarchy enhances informational content in the network. Through Hebbian-like mechanisms, it may facilitate both memory formation and activation when a match is made between incoming sensory information and a memory trace (Brefczynski-Lewis and Lewis, 2017). This enhancement of informational content is also consistent with the integrated information theory of consciousness that places integrated information at the center of conscious experience (Seth and Bayne, 2022). Importantly, the integrated information theory links consciousness with neural activity within posterior (parietal, temporal, and occipital) cortical areas, while the global neuronal workspace theory places an emphasis on activation of prefrontal cortex. In the present study, prominent behavior-related activity was observed in posterior (left AG and MOG) as well as prefrontal regions (IFGtr, MFG, OG), providing support for elements of both the global neuronal workspace and integrated information frameworks.

### 4.6. Caveats

A key concern applicable to all human iEEG studies performed in epilepsy patients is the degree to which such data can be extrapolated to the general population (Yang and Wang, 2023). Effects of epilepsy and possible secondary reorganization of brain regions outside diagnosed seizure foci (diaschisis), as well as potential effects of antiseizure medications, are common to all human iEEG studies (Parvizi and Kastner, 2018; Grewe and Bien, 2023). To address this concern, all analyses were preceded by establishing consistency with results of previous studies of auditory cortex (reviewed in Nourski, 2017).

While this study was based on a large cohort of 49 participants with a total of 6744 recording sites examined, we were not able to characterize the balance between stimulus- and behavior-related activity in some ROIs (see Table 1 and Fig. 4). This was due to the limited high gamma activation in either post-stimulus or pre-behavior window at recording sites in these ROIs. This limited extent of activation is likely secondary to the choice of the experimental paradigm, including both the stimuli and the task (semantic categorization of single monosyllabic words with button press behavioral responses). Indeed, the degree to which the present findings can be generalized to auditory speech processing may be limited by the choice of task, specific semantic categories used, as well as the recording modality (Santoro et al., 2014; Brefczynski-Lewis and Lewis, 2017; Bracci et al., 2017). For instance, we did not examine semantic processing at the sentence level, which could be expected to strongly activate the anterior temporal lobe (Dronkers et al., 2004).

The choice of non-overlapping time windows used to define stimulus- and behavior-related activity patterns was dictated by the relatively short RTs due to the absence of a delayed response cue. Still, neural activity that was temporally related to the stimulus and to the behavioral response likely had some degree of overlap at individual recording sites. While a response cue would better separate neural activity associated with stimulus and behavior, the absence of a forced delay provides for a more naturalistic experimental setting where the behavior is self-initiated. The definition criteria for stimulus- and behavior-related patterns (significant ERBP in one window but not the other) were conservative, and the third, intermediate, pattern may represent prolonged stimulus-driven activation or early-onset behavior-related activity to varying degrees.

Finally, the present study specifically focused on correct hit trials and did not examine behavior-related activity associated with FA responses or stimulus-related activity during miss trials. Due to the relatively simple nature of the task, most participants had very few FA and miss trials (see Supplementary Table 1), precluding analyses of task performance effects across the entire participant cohort.

### 4.7. Future directions

This study examined stimulus representation and behavior-related activity using a speech stimulus-based task. It remains to be determined whether the same findings, particularly hemispheric differences and auditory stimulus-driven activity within sensorimotor cortex, would generalize to non-speech stimuli. Additionally, it would be of interest to examine the regional distribution of behavior-related activity in tasks involving visual stimuli or verbal responses (Rhone et al., 2016; Van Engen et al., 2022; Tiippana, 2023; Yu et al., 2025) and establish the degree of spatial overlap with the results of the present study. While the current work focused on high gamma band activity, future complementary analyses examining alpha and beta-band activity and suppression may shed light on the interplay between feedforward and feedback signaling during semantic classification tasks (Nourski et al., 2021, 2024b; Ono et al., 2023). Future studies involving effective connectivity analyses (e.g., Kitazawa et al., 2025) may further clarify functional relationships between stimulus- and behavior-locked activity.

## Supplementary Material

1

Supplementary materials

Supplementary material associated with this article can be found, in the online version, at doi:10.1016/j.neuroimage.2026.121801.

## Figures and Tables

**Table 1:** Region of interest (ROI) parcellation scheme and abbreviations.

ROI group	ROI abbreviation	ROI
Auditory core	HGPM	Heschl’s gyrus, posteromedial portion
Superior temporal plane (STP)	HGAL	Heschl’s gyrus, anterolateral portion
	PP	Planum polare
	PT	Planum temporale
Superior temporal gyrus (STG)	STGM	Superior temporal gyrus, middle portion
	STGP	Superior temporal gyrus, posterior portion
Ventra	MTGA	Middle temporal gyrus, anterior portion
	MTGM	Middle temporal gyrus, middle portion
	MTGP	Middle temporal gyrus, posterior portion
	STGA	Superior temporal gyrus, anterior portion
	STSL	Superior temporal sulcus, lower bank
	STSU	Superior temporal sulcus, upper bank
Dorsal	AG	Angular gyrus
	SMG	Supramarginal gyrus
Limbic	Amyg	Amygdala
	Hipp	Hippocampus
	PHG	Parahippocampal gyrus
Prefrontal	ACC	Anterior cingulate cortex
	FP	Frontal pole
	GR	Gyrus rectus
	IFGop	Inferior frontal gyrus, pars opercularis
	IFGor	Inferior frontal gyrus, pars orbitalis
	IFGtr	Inferior frontal gyrus, pars triangularis
	MFG	Middle frontal gyrus
	OG	Orbital gyri
	SFG	Superior frontal gyrus
	SubcG	Subcallosal gyrus
Sensorimotor	ParaCL	Paracentral lobule
	PostCG	Postcentral gyrus
	PreCG	Precentral gyrus
Other	CingMA	Cingulate cortex, middle anterior portion
	CingMP	Cingulate cortex, middle posterior portion
	CingPD	Cingulate cortex, posterior dorsal portion
	Cun	Cuneus
	FG	Fusiform gyrus
	IOG	Inferior occipital gyrus
	ITGA	Inferior temporal gyrus, anterior portion
	ITGM	Inferior temporal gyrus, middle portion
	ITGP	Inferior temporal gyrus, posterior portion
	InsA	Anterior insula
	InsP	Posterior insula
	LingG	Lingual gyrus
	MOG	Middle occipital gyrus
	OP	Occipital pole
	PMC	Premotor cortex
	PreCun	Precuneus
	SOG	Superior orbital gyrus
	SPL	Superior parietal lobule
	TP	Temporal pole
	VStr	Ventral striatum
	POp	Parietal operculum

**Table 2:** Hemispheric distribution of stimulus-related, intermediate and behavior-related patterns across ROI groups.

ROI group	Stimulus-related			Intermediate			Behavior-related		
	Left	Right	*p*	Left	Right	*p*	Left	Right	*p*
HGPM	97/100	77/78	0.893	1/100	1/78	1	0/100	0/78	1
STP	96/165	82/138	1	10/165	12/138	0.771	1/165	0/138	1
STG	157/295	138/228	0.292	32/295	14/228	0.253	1/295	2/228	0.893
Ventral	66/574	89/593	0.290	15/574	19/593	0.893	15/574	5/593	0.140
Dorsal	28/264	48/254	0.0715	6/264	8/254	0.893	7/264	4/254	0.893
Limbic	3/211	1/214	0.771	3/211	2/214	0.912	10/211	9/214	0.982
Prefrontal	26/765	25/675	0.981	6/765	12/675	0.292	60/765	24/675	**0.0133**
Sensorimotor	32/332	49/257	**0.0133**	18/332	5/257	0.158	13/332	5/257	0.548

**Table 3:** Summary of stimulus/behavior index (“SB index”) measurements across ROIs for both hemispheres.

ROI group	ROI	Left hemisphere					Right hemisphere				
		*n*_total_	*n*_stim_	*n*_behav_	*N*	SB index	*n*_total_	*n*_stim_	*n*_behav_	*N*	SB index
Auditory core	HGPM	100	97	0	26	−1	78	77	0	21	−1
STP	HGAL	63	45	0	18	−1	46	35	0	13	−1
	PP	45	1	1	2	—	52	9	0	5	−1
	PT	57	50	0	17	−1	40	38	0	14	−1
STG	STGM	115	46	0	12	−1	100	42	1	12	−0.953
	STGP	180	111	1	19	−0.982	128	96	1	15	−0.979
Ventral	MTGA	88	4	4	5	0	57	3	0	2	—
	MTGM	133	10	2	7	−0.667	157	17	1	11	−0.889
	MTGP	162	20	2	11	−0.818	196	27	1	11	−0.929
	STGA	39	0	2	2	—	29	0	2	2	—
	STSL	109	26	2	9	−0.857	113	23	1	13	−0.917
	STSU	43	6	3	6	−0.333	41	19	0	8	−1
Dorsal	AG	95	1	5	4	0.667	106	12	1	5	−0.846
	SMG	169	27	2	11	−0.862	148	36	3	9	−0.846
Limbic	Amyg	74	1	5	4	0.667	84	0	3	1	—
	Hipp	67	2	3	5	0.200	80	1	4	2	−0.600
	PHG	70	0	2	2	—	50	0	2	2	—
Prefrontal	ACC	22	0	0	0	—	28	0	0	0	—
	FP	30	0	1	1	—	31	0	2	2	—
	GR	38	1	1	2	—	34	4	1	4	−0.600
	IFGop	71	8	6	8	−0.143	41	5	2	6	−0.429
	IFGor	16	0	0	0	—	15	1	0	1	—
	IFGtr	98	3	14	11	0.647	89	6	3	6	−0.333
	MFG	218	6	22	14	0.571	194	4	8	7	0.333
	OG	182	7	12	10	0.263	192	4	8	10	0.333
	SFG	79	1	3	3	—	49	1	0	1	—
	SubcG	12	0	2	2	—	2	0	0	0	—
Sensorimotor	ParaCL	2	1	0	1	—	7	1	0	1	—
	PostCG	146	15	4	10	−0.579	103	28	2	10	−0.867
	PreCG	184	16	9	13	−0.280	147	20	3	12	−0.739
Other	CingMA	26	0	1	1	—	15	0	0	0	—
	CingMP	13	0	0	0	—	9	0	0	0	—
	CingPD	16	0	0	0	—	7	1	0	1	—
	Cun	10	1	0	1	—	6	1	0	1	—
	FG	94	4	4	5	0	92	0	0	0	—
	IOG	11	0	0	0	—	12	0	0	0	—
	ITGA	76	1	3	4	—	61	0	2	2	—
	ITGM	68	2	2	3	—	47	1	0	1	—
	ITGP	52	2	1	2	—	46	2	1	3	—
	InsA	71	5	1	4	−0.667	41	1	0	1	—
	InsP	74	36	2	15	−0.895	51	27	0	14	−1
	LingG	28	0	0	0	—	15	3	0	2	—
	MOG	73	2	6	4	0.500	23	0	0	0	—
	OP	3	0	0	0	—	7	0	0	0	—
	PMC	89	5	7	8	0.167	59	3	0	3	—
	PreCun	19	0	0	0	—	15	0	0	0	—
	SOG	13	2	0	1	—	1	0	0	0	—
	SPL	25	0	3	3	—	17	0	0	0	—
	TP	144	1	5	4	0.667	126	3	3	5	0
	VStr	16	1	0	1	—	10	0	0	0	—
	POp	15	4	0	3	—	4	0	0	0	—

**Table 4:** Relationship between stimulus-related high gamma activity and RTs across ROIs.

ROI group	ROI	*n*_sites_	median rho	*p*
HGPM	HGPM	176	− 0.0271	0.0617
STP	HGAL	86	− 0.0356	**0.0276**
	PP	20	− 0.00657	0.657
	PT	94	− 0.0222	0.154
STG	STGM	104	− 0.0463	**0.00230**
	STGP	237	− 0.0603	**<0.0001**
Ventral	MTGM	30	0.0668	0.125
	MTGP	63	0.0378	0.261
	STSL	56	0.0248	0.106
	STSU	32	− 0.0289	0.691
Dorsal	AG	16	− 0.0952	0.0858
	SMG	74	− 0.0361	0.093
Prefrontal	IFGop	17	− 0.140	**0.0329**
	IFGtr	15	− 0.0723	0.116
	MFG	16	− 0.0822	**0.0276**
	OG	11	− 0.00655	1.00
Sensorimotor	PostCG	49	− 0.0156	0.891
	PreCG	53	− 0.0217	0.138
Other	InsP	64	− 0.0152	0.770
	PMC	15	− 0.104	**0.0102**

## Data Availability

Data and code are available upon request to the Corresponding Author pending establishment of a formal data sharing agreement.
