# Supplementary material for: Stimulus-driven and behavior-driving activity along the cortical auditory hierarchy
Source: Neuroimage. Author manuscript; Available in PMC 2026 Mar 11. (PMC12977194; doi:10.1016/j.neuroimage.2026.121801)
Supplement: 1 [file NIHMS2151683-supplement-1.docx]

***Supplementary Material***

**Stimulus-driven and Behavior-driving Activity along the Cortical Auditory Hierarchy**

Kirill V. Nourski^1,2^*, Mitchell Steinschneider^1,3^*, Ariane E. Rhone^1^, Matthew A. Howard III^1,2,4^

*^1^Department of Neurosurgery, The University of Iowa, Iowa City, IA 52242, USA
^2^Iowa Neuroscience Institute, The University of Iowa, Iowa City, IA 52242, USA
^3^Departments of Neurology, Neuroscience, and Pediatrics, Albert Einstein College of Medicine,
Bronx, NY 10461, USA
^4^Pappajohn Biomedical Institute, The University of Iowa, Iowa City, IA 52242, USA*

**Equally contributed to this work*

**Supplementary Table 1.** Participant demographics, electrode coverage and task performance.

| **Participant** | **Age (years)** | **Sex^2^** | **Handedness^1^** | **Language dominance^1^** | **Subdural arrays^3^** | ***n*_sites_^4^** | **Hit rate (%)** | ***d*’** | **Median RT (s)** | **Fast hits (RT<0.55 s) (%)** |
| --- | --- | --- | --- | --- | --- | --- | --- | --- | --- | --- |
| L258 | 38 | M | R | L | Y | 176 | 83.8 | 3.33 | 0.827 | 1.49 |
| L275 | 30 | M | R | L | Y | 222 | 67.8 | 3.21 | 0.868 | 1.64 |
| R288 | 21 | M | R | L | Y | 198 | 84.4 | 3.42 | 0.814 | 0 |
| L292 | 50 | F | L | R | Y | 157 | 82.2 | 3.07 | 0.837 | 6.76 |
| L307 | 29 | M | R | L | Y | 208 | 96.7 | 4.09 | 0.858 | 0 |
| L314 | 31 | F | R | L | Y | 59 | 24.4 | 1.16 | 1.307 | 0 |
| R316 | 31 | F | R | L | Y | 95 | 91.1 | 4.00 | 0.637 | 15.9 |
| R320 | 51 | F | R | L | Y | 230 | 98.8 | 4.67 | 0.721 | 8.33 |
| R322 | 29 | F | R | ? | Y | 79 | 90.0 | 3.34 | 0.831 | 1.23 |
| R334 | 39 | M | L | L | Y | 230 | 74.4 | 2.63 | 0.937 | 1.49 |
| B335 | 33 | M | R | L | Y | 144 | 98.9 | 4.54 | 0.615 | 24.7 |
| L357 | 37 | M | R | L | Y | 158 | 40.0 | >5 | 0.838 | 5.56 |
| R369 | 30 | M | R | L | Y | 206 | 91.1 | 3.49 | 0.683 | 15.9 |
| L372 | 34 | M | R | L | Y | 188 | 86.7 | 3.52 | 0.656 | 8.97 |
| R376 | 48 | F | R | L | Y | 208 | 46.7 | 1.27 | 0.762 | 4.76 |
| R399 | 22 | F | R | L | Y | 185 | 80.0 | 2.82 | 1.026 | 0 |
| L405 | 19 | M | R | L | Y | 140 | 51.1 | 1.73 | 1.226 | 0 |
| L409 | 31 | F | R | L | Y | 170 | 93.3 | 3.55 | 0.739 | 8.33 |
| L416 | 34 | M | B | L | Y | 136 | 38.9 | 1.05 | 1.275 | 8.57 |
| R418 | 25 | F | R | L | Y | 87 | 94.4 | 4.25 | 0.841 | 2.35 |
| L423 | 51 | M | R | L | Y | 177 | 72.2 | 2.39 | 1.137 | 1.54 |
| R429 | 32 | F | R | L | Y | 156 | 76.7 | 2.39 | 1.049 | 10.1 |
| R456 | 31 | M | L | L | Y | 207 | 86.7 | 3.52 | 0.819 | 1.28 |
| B457 | 18 | M | R | L | N | 112 | 73.3 | 2.53 | 0.847 | 4.55 |
| L460 | 52 | M | R | L | Y | 163 | 93.3 | 3.76 | 0.889 | 0 |
| L477 | 23 | F | R | L | Y | 125 | 90.0 | 3.26 | 0.807 | 1.23 |
| L514 | 46 | M | R | L | N | 122 | 75.6 | 2.75 | 1.074 | 1.47 |
| L525 | 46 | F | R | L | Y | 231 | 97.8 | 4.66 | 0.852 | 0 |
| R532 | 42 | F | R | L | Y | 193 | 84.4 | 2.64 | 0.782 | 5.26 |
| R561 | 19 | M | R | L | N | 149 | 88.9 | 3.37 | 0.905 | 0 |
| R567 | 33 | M | R | ? | N | 93 | 24.4 | 1.45 | 1.02 | 0 |
| L585 | 39 | F | R | L | Y | 157 | 93.3 | 3.91 | 0.806 | 0 |
| L625 | 24 | F | R | L | N | 88 | 82.2 | 2.78 | 0.700 | 17.6 |
| L634 | 22 | F | R | L | N | 77 | 98.9 | 4.94 | 0.705 | 2.25 |
| L640 | 45 | F | R | B | N | 57 | 98.9 | 4.94 | 0.621 | 21.3 |
| R672 | 36 | F | R | L | N | 85 | 96.7 | >5 | 0.680 | 17.2 |
| L702 | 22 | M | R | B | N | 113 | 86.7 | 3.19 | 0.702 | 1.28 |
| R717 | 55 | M | L | L | N | 106 | 100 | >5 | 0.851 | 4.44 |
| R720 | 42 | F | R | L | N | 72 | 64.4 | 2.11 | 0.666 | 20.7 |
| R728 | 40 | M | R | L | N | 114 | 98.9 | 4.72 | 0.535 | 53.9 |
| L741 | 44 | F | R | L | N | 100 | 98.9 | 4.30 | 0.546 | 52.8 |
| R764 | 32 | F | B | L | N | 75 | 88.9 | 3.23 | 0.781 | 8.75 |
| L789 | 48 | M | R | B | N | 73 | 96.7 | 3.84 | 0.730 | 2.30 |
| L820 | 51 | M | R | L | Y | 196 | 91.1 | 3.52 | 0.921 | 1.22 |
| L841 | 56 | F | R | L | N | 70 | 85.6 | 2.72 | 0.962 | 0 |
| R846 | 32 | F | R | L | N | 79 | 93.3 | 3.79 | 0.656 | 13.1 |
| L855 | 33 | M | R | L | N | 99 | 96.7 | >5 | 0.883 | 0 |
| L857 | 39 | M | R | L | N | 76 | 77.8 | 2.33 | 0.601 | 17.1 |
| B891 | 35 | M | R | L | N | 103 | 98.9 | 4.72 | 0.719 | 6.74 |

^1^L: left; R: right; B: bilateral; ?: undetermined.
^2^F: female; M: male.
^3^Y: yes; N: no.
^4^Number of recording sites examined in the present study (excluding recording sites identified as seizure foci, characterized by excessive noise, and depth electrode contacts in white matter or outside the brain).

*
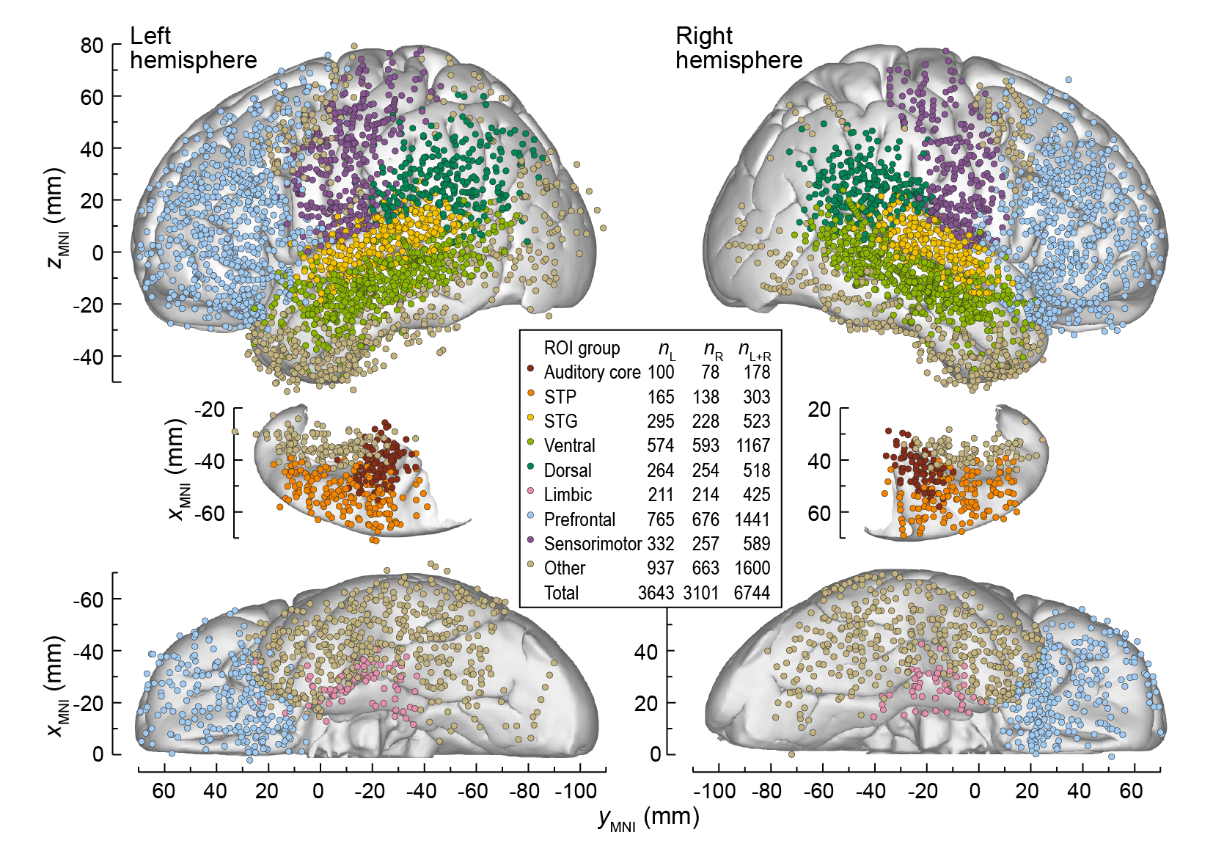
*

**Supplementary Figure 1.** Summary of electrode coverage for all 49 participants. Locations of recording sites, determined for each participant individually and color-coded by region-of-interest (ROI) group, are plotted in MNI coordinate space and projected onto the Freesurfer average template brain for spatial reference. Projections are shown in the lateral, top-down (superior temporal plane), and ventral views (top to bottom rows). Numbers of sites in each ROI group across all participants in the left (*n*_L_), right (*n*_R_) hemisphere and in both hemispheres (*n*_L+R_) are denoted in the legend. Sites in the amygdala, hippocampus, cingulate, subcallosal, lingual gyrus, paracentral lobule, cuneus, precuneus, ventral striatum and parietal operculum are not shown but were analyzed.
